# Pharmacological inhibition of the canonical Wnt/β-catenin signaling pathway sensitizes 5-fluorouracil-resistant colorectal cancer cells

**DOI:** 10.1007/s11033-026-12711-z

**Published:** 2026-09-04

**Authors:** Diego Alfonso Arregui Ramos, Daniela Filomena Tavares de Pina, Annie Cristhine Moraes Sousa-Squiavinato, Wallace Martins de Araújo, Murilo Ramos Rocha, Jose Andres Morgado-Diaz

**Affiliations:** 1 Cellular and Molecular Oncobiology Program, Brazilian National Cancer Institute (INCA), 37 André Cavalcanti St, 3th Floor, Rio de Janeiro, RJ 20230-051 Brazil; 2 Present Address: Annie Cristhine Moraes Sousa-Squiavinato, Immunology and Tumor Biology Program, Brazilian National Cancer Institute (INCA), Rio de Janeiro, Brazil; 3 Present Address: Department of Biochemistry and Molecular Biology, Johns Hopkins Bloomberg School of Public Health, Baltimore, MD USA

**Keywords:** Colorectal cancer, Wnt/β-catenin pathway, Cancer stem cells, 5-fluorouracil, LF3

## Abstract

**Background:**

The activation of the Wnt/β-catenin pathway with chemoresistance in colorectal cancer has been hypothesized. However, the use of specific inhibitors for targeting this pathway has not been well explored. In the present study, we analyze the activation of this pathway and its role in the stemness phenotype acquisition to regulate chemoresistance using our long-term 5-fluorouracil (5-FU) ‐ resistant model of colorectal cancer cells, known to present epithelial-mesenchymal transition and enhanced migration and invasion.

**Methods and results:**

Initially by bioinformatic analyses we demonstrate the association between 5-FU resistance genes with those of a stem cell-like phenotype in patients with colon cancer. In addition, 5-FU-resistant cells displayed stemness characteristics, with upregulation of key stem cell marker (*ALDH1A1*) using reverse transcription-quantitative polymerase chain reaction. Further, 5-FU-resistant cells exhibited high Wnt/β-catenin pathway activity. Interestingly, treatment with LF3, an inhibitor of this pathway, re-sensitized the 5-FU-resistant cells, decreasing their proliferation.

**Conclusion:**

Wnt/β-catenin pathway activation plays a role in regulating key cellular events involved in chemoresistance. The study findings suggest the use of combinatorial therapies using conventional agents, such as 5-FU, and inhibitors of this pathway, as a useful therapeutic strategy to treat patients with colorectal cancer.

## Introduction

Colorectal cancer (CRC) ranks third in global incidence and is the second leading cause of cancer-related mortality, with an alarming increase in incidence among individuals below 50 years of age [1, 2]. The 5-year survival rate is low yet, despite improvement in treatment strategies, since a subset of patients experience recurrence or distant metastasis due to therapy resistance mechanisms [3, 4]. Therefore, it is necessary to develop new treatment strategies that select tumor vulnerabilities to improve patient prognosis.

Previous CRC studies have suggested potential association of the Wnt/β-catenin signaling with therapeutic failure, which justifies further elucidation of its regulatory mechanism for successful application in cancer therapy. When activated, this pathway promotes the renewal, proliferation, and differentiation in cancer stem cells (CSCs), as well as epithelial-mesenchymal transition (EMT), promoting the metastatic dissemination of cancer cells [5, 6]. Owing to its involvement in crucial steps of cancer progression, the dynamics of this pathway and its components have been hypothesized as potential targets for CRC and other different cancer types [7–10].

Wnt/β-catenin signaling overexpression can originate from mutations in the β-catenin gene or those genes encoding members of the destruction complex, as well as from the overexpression of Wnt ligands, Frizzled receptors and Dkk-1 protein leading to malignancy [11, 12]. In addition, its relationship with chemotherapy resistance in CRC has also been hypothesized [13]. Recently, we developed a long-term 5-fluorouracil-resistant CRC cell (HCT-116 5FUR) model and demonstrated that, in comparison with its parental cell line, these cells displayed EMT features with enhanced migration and invasion capabilities. The resistance gene signature to 5-FU was associated with the transition epithelial-mesenchymal signature as *ABCB1* and *ZEB2* were highly co*-*expressed in patients with CRC [14]. However, Wnt/β-catenin signaling activation status in HCT-116 5FUR and its role in the stemness phenotype to regulate chemoresistance, was not evaluated.

Therefore, in the present study we determine if the Wnt/β-catenin pathway activation is associated with CSC features as a resistance mechanism to 5-FU. Furthermore, we aimed to analyze the small molecule, LF3, which blocks the interaction between β-catenin and the Transcription Factor 4 (TCF4), thus interfering with the activation of this pathway, as a sensitizer to 5FUR cells.

## Materials and methods

### Cell lines

**Fig. 1:**
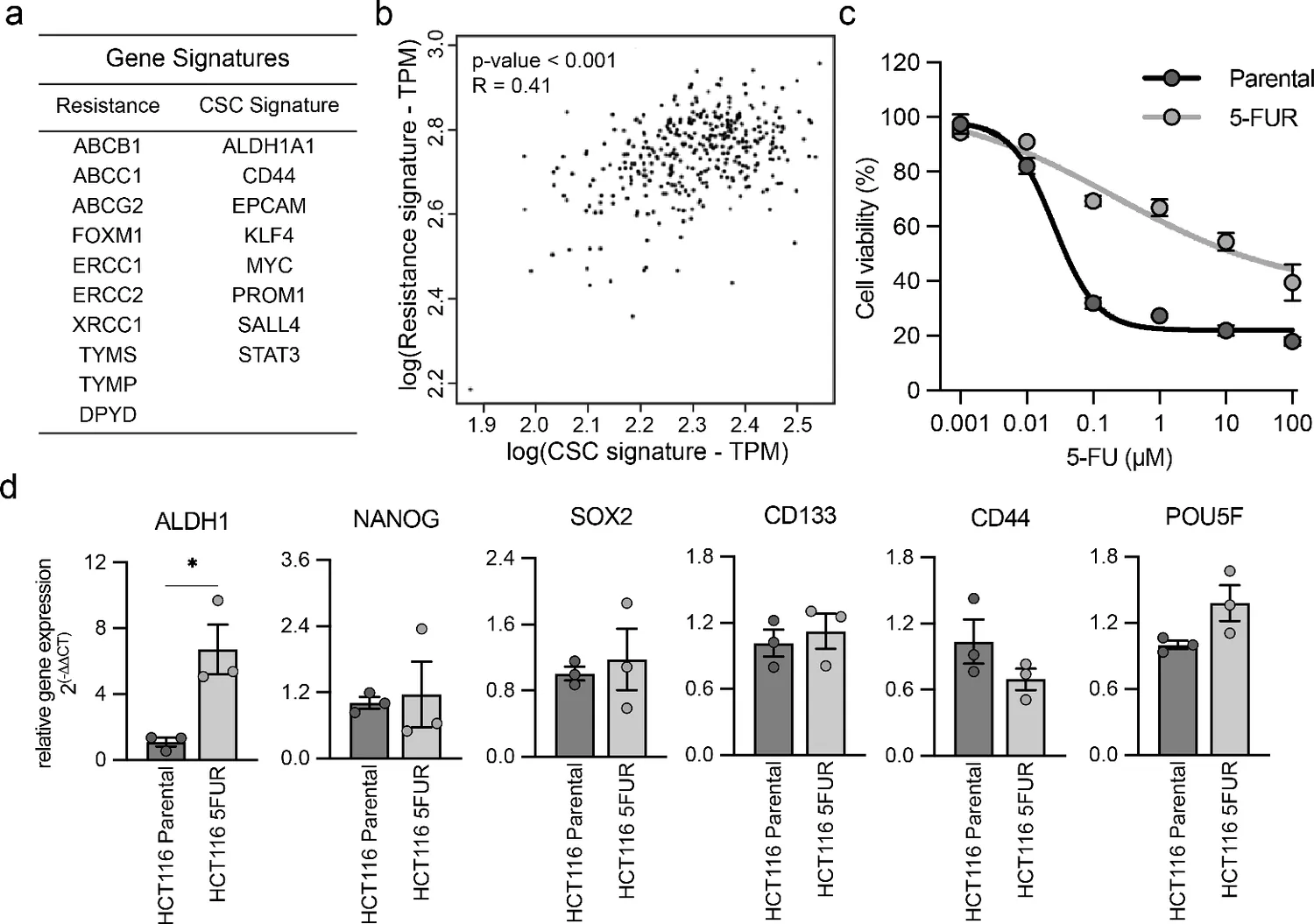
Analysis of gene signatures, correlation between 5-fluorouracil (5-FU) chemoresistance and cancer stem cell (CSC) phenotype (**a**) Gene signatures related to 5-FU resistance and the CSC phenotype. (**b**) Correlation between the gene signature of chemoresistance to 5-FU and the CSC phenotype based on Pearson’s coefficient using the online bioinformatics tool, GEPIA 2 (http://gepia2.cancerpku.cn; 2023). (**c**) Cell viability in HCT 116 control and thawed HCT 116 5FUR cells using the MTT assay after treatment for 48 h with increasing 5-FU concentrations. Results shown were independently repeated during the revision process. (**d**) Reverse transcription-quantitative polymerase chain reaction analysis of markers associated with the CSC phenotype. The 18 S gene was used as an internal control for gene expression. Data are representative of the mean ± standard error of mean (SEM) of three independent experiments. Statistical analysis was performed using Student’s t-test. The difference was considered significant when *P < 0.05. TPM, Transcripts per million

For this study, we used the long-term 5-fluorouracil (5-FU)-resistant CRC cell (HCT-116 5FUR) model obtained in our laboratory as previously reported [14]. HCT-116 cells used to obtain this model were purchased from the American Type Culture Collection (ATCC, USA) and the origin and identity of the cell line were confirmed by short tandem repeat (STR) analysis. Prior to experiment initiation, the cells were thawed and assessed for the maintenance of the resistant phenotype by treating the cells with increasing 5-FU concentrations (0.01–100 µM), and the cell viability was determined. Viability curve in Fig. 1c was repeated independently during the review process and the corresponding results are shown.

### Antibodies and reagents

Anti- glycogen synthase kinase 3 beta (GSK-3β) and anti-pGSK-3β S9 antibodies were obtained from Cell Signaling (Danvers, MA, USA); anti-cyclin D1 was purchased from Santa Cruz (Dallas, TX, USA); anti-α-Tubulin was obtained from Sigma Aldrich (Burlington, MA, USA); and anti-glyceraldehyde 3-phosphate dehydrogenase (GAPDH) was obtained from Ambion (Carlsbad, CA, USA). The mouse anti-β-catenin was obtained from Thermo Fisher (Waltham, MA, USA), and the anti-active β-catenin (anti-ABC) was obtained from Merck Millipore (Burlington, MA, USA). The secondary antibody Alexa Fluor^®^ 546 IgG-goat-anti-mouse was purchased from Molecular Probes (Eugene, Oregon, USA) and the horseradish peroxidase-conjugated anti-mouse and anti-rabbit IgG antibodies were purchased from Sigma-Aldrich (St. Louis, MO, USA). The Wnt/β-catenin pathway inhibitor, LF3, was obtained from MedChemExpress (Monmouth Junction, NJ, USA). The 5-FU (Eurofarma, RJ, Brazil) was a kindness of the Pharmacy Service of the Cancer Hospital I - Bone Marrow Transplant Center - INCA-HC1/RJ.

### LF3 treatment

For LF3 treatment, intermediate dilutions at concentrations of 10, 20, 30, 40, and 60 µM were prepared from the 10 mM stock solution. The dilutions were made using DMEM culture medium, and the cells were treated for 24–48 h, depending on the experimental protocol.

### Cell viability assay

Cell viability was assessed using the MTT (Sigma Aldrich) assay. Cells were plated at a density of 2 × 10⁴ cells in 96-well plates and treated with increasing 5-FU concentrations for 48 h. Then, the cells were treated with the MTT 3-(4,5-dimethylthiazol-2-yl)−2,5-diphenyltetrazolium bromide) reagent for 2 h at 37 °C protected from light and eluted with DMSO. Absorbance was measured at 492 nm using a SpectraMax 190 ID3 (Molecular Devices, Sunnyvale, CA, USA). The inhibitory concentration values corresponding to the 50% rate (IC50) were calculated from dose-response curves, relating the cell viability of parental and resistant cells under different concentrations of the drug using GraphPad Prism 8.0 software (San Diego, CA, USA). The experiments were performed in triplicate and at least three times.

### Western blotting

Cell lysate preparation, sodium dodecyl sulfate–polyacrylamide gel electrophoresis, protein transfer as well as blocking were done as previously described [15]. Afterwards, the membranes were incubated for 12 h with the following primary antibodies: anti-β-catenin (1:1000), anti-active β-catenin (1:1000), anti-GSK3β (1:1000), anti-pGSK3β S9 (1:1000), anti-cyclin D1 (1:1000), anti-α-Tubulin (1:1000), and anti-GAPDH (1:50.000). Then, washed membranes were incubated for 1 h with the corresponding secondary antibodies conjugated to peroxidase, at a dilution of 1:10000. Labeling was revealed using the enhanced chemiluminescence (ECL) kit (Amersham Biosciences GE Healthcare UK Limited, Buckinghamshire, UK) and the ChemiDoc™ Imaging System (Bio-Rad Laboratories, Hercules, CA, USA).

### Immunofluorescence

Cells were seeded on coverslips into 12-well plates and incubated for 24 h at 37 °C. The cells were washed three times with phosphate-buffered saline (PBS) and fixed with 4% paraformaldehyde in PBS for 20 min at 24 °C Subsequently, the fixed cells were washed and permeabilized with 0.4% Triton X-100 for 20 min at 24 °C. Non-specific sites were saturated with 1% bovine serum albumin (BSA) for 20 min. The cells were then incubated by 12 h, at 4 °C with the primary antibodies: total β-catenin (1:250) and active β-catenin (1:30) diluted in 1% BSA. Afterwards, the cells were washed and incubated for 1 h, at 24 °C with secondary antibodies conjugated to Alexa Fluor 546 (1:250). The cells were again washed and incubated with 4′,6-diamidino-2-phenylindole 4′,6-diamidino-2-phenylindole (DAPI); 1:1000 for 1 min. Finally, the slides were mounted in a solution of Antifade Gold ProLong reagent (Invitrogen, Carlsbad, CA, USA) and images acquired using an AXIO OBSERVER.Z1 microscope (Carl Zeiss, Germany). Contrast and colors were standardized with an ICY bioimage analysis software. (Institut Pasteur, Paris – France).

### Transcriptional activity of β-catenin

The transcriptional activity of β-catenin was measured using the T-cell factor (TCF)/Lymphoid enhancer factor (LEF) transcription factor reporter activity with a Dual-Luciferase Reporter Assay System Kit (Promega, Madison, WI, USA) as previously reported [16]. Briefly, cells were placed in 12-well plates at a density of 2 × 10⁴ cells and transfected with the M50 Super8x TOPflash plasmid or the mutated M51 Super8X FOPflash plasmid (Addgene, Watertown, MA, USA), which serves as a negative control for activity. Cells were transiently co-transfected with 2 µg of PRL-TK Renilla luciferase (Promega, Madison, WI, USA). Transfection was performed using the FUGENE Transfection Reagent (Roche Applied Science, Basel, Switzerland) according to the suppliers’ instructions. After a 24 h transfection period, the cells were washed, lysed, and Renilla and Luciferase activities were measured using the Dual Luciferase Reporter Assay System Kit (Promega, Madison, WI, USA). The reading was performed using a Verita plate luminometer (Tuner Biosystems, Sunnyvale, CA, USA). Luciferase activity was normalized by Renilla Luciferase activity.

### RT-qPCR

Total RNA was extracted using the Trizol^®^ method (Invitrogen, Carlsbad, CA, USA) from 2 × 10⁶ cells, and the cDNA synthesis was performed using the Mix Impro II kit (Promega, Madison, WI, USA) from 1 µg of RNA for each sample. The quantification of gene expression of the selected genes was performed using the polymerase chain reaction technique and quantitative real-time polymerase chain reaction (qPCR) using SYBR Green^®^ PCR Master Mix39 (Invitrogen, Carlsbad, CA, USA) in a Vii 7 real-time PCR instrument (Thermo Scientific). In each reaction, 200–500 ng of final cDNA concentration from the samples, 5.0 µL of SYBR Green^®^ reagent, and primers at a final concentration of 0.2 µM in a volume of 2.5 µL were used. The cycling profiles were as follows: 40 cycles of 95 °C–30 s/60 °C–30 s/72 °C–30 s. The primer sequences used in this study were:

*CD44* F 5′-CTTCAGGTTACATCTTTTACA-3′, *CD44* R 5′-TCATCAAAGTGGTAGCAGGGA-3′;

*PROM1* F 5′-TCATACTGGTGGCTGGGTGG-3′, *PROM1* R 5′-GGTGGTCGGGGTGGCAT-3′;

*ALDH1* F 5′-TGCTGGGCACAATGGAGTCAATG-3′, *ALDH1* R 5′-AACCTGCACAGTAGCGCAATGT-3′;

*SOX2* F 5′-TACAGCATGTCCTACTCGCAG-3′, *SOX2* R 5′-GAGGAAGAGGTAACCACAGGG-3′.

*POU5F1* (OCT4) F 5′-TGGTCCGAGTGTGGTTCTGTAA-3′, *POU5F1* (OCT4) R 5′-TGTGCATAGTCGCTGCTTGAT-3′;

*NANOG* F 5′-AGTCCCAAAGGCAAACAACCCA-3′, *NANOG* R 5′-TGCTGGAGGCTGAGGTATTCCTGTC-3′;

18 S F 5′-AACCCGTTGAACCCCATT-3′, 18 S R 5′-CCATCCAATCGGTAGTAGCG-3′.

The cycle threshold (CT) values of the analyzed transcripts were normalized based on concomitant analysis with those of the gene encoding the 18 S protein. The calculation of the differential expression of the selected genes was performed using the 2^-ΔΔCt^ method.

### Correlation between 5-FU resistance and CSC signatures

We performed correlation analyses using the GEPIA2 database (http://gepia2.cancer-pku.cn/#about) based on colon cancer (COAD, *n* = 275) data from The Cancer Genome Atlas database as described by us [14]. Genes of 5-FU resistance and those related to CSC phenotype were selected, and correlation analyses were done using Pearson’s method on the GEPIA2 web server.

### Statistical analyses

The GraphPad Prism 8.0 program (GraphPad™ Software, San Diego, CA, USA) was used to calculate the significance of the observed differences between the studied conditions, as well as to construct the graphs. For the comparison between two groups with normal distribution, the Student’s t-test was used. In the case of comparisons between three or more groups, the one-way analysis of variance followed by Tukey’s Post Hoc test. Differences were considered significant when: *P < 0.05, **P < 0.01, ***P < 0.001, ****<0.0001. Data are representative of the mean ± standard error of the mean of three independent experiments.

## Results and discussion

In the present study, we assess the involvement of the Wnt/β-catenin pathway during chemoresistance acquisition. To achieve this, we used our long-term 5FU‐resistant HCT-116 5FUR model, wherein the cells were characterized by factors such as the development of EMT characteristics. Here, we analyzed the Wnt/β-catenin pathway activation status and its association with CSC behaviors. In addition, we evaluated the sensitization of these cells to the LF3 inhibitor, which blocks the interaction between β-catenin and TCF4, thus hindering its activation.

### Association between CSCs and 5-FU resistance gene signatures in samples from patients with CRC

Initially, bioinformatic analysis was used to determine the relation between the CSC gene signature and the 5-FU resistance gene signature in samples from patients with CRC. For this purpose, we used transcript data from patients with colon cancer available on the public GEPIA platform. Genes related to 5-FU resistance, DNA damage repair, and 5-FU metabolism were selected. The gene signatures associated with CSCs included the genes *CD44*, prominin 1 (*PROM1*), *EPCAM* (transmembrane glycoproteins), kruppel-like factor 4 (*KLF4*), *MYC*, *STAT3*, spalt-like transcription factor 4 (*SALL4*) (transcription factors) and *ALDH1A1* (detoxifying enzyme) [17] (Fig. 1a). Interestingly, a significant and positive correlation (*R* = 0.41, *p* < 0.001) was observed between the gene signatures of resistance to 5-FU and CSCs (Fig. 1b). These results suggest an association between genes of 5-FU resistance and those related to the CSC phenotype in patients with colon cancer. Figure 1c cell viability assay confirms the 5-FU chemoresistant phenotype of the HCT-116 5FUR cells previously obtained in our laboratory, showing that after cryo-recovery and challenged with increasing 5-FU concentrations these cells maintained the resistant phenotype. Considering that both EMT and stemness are known to be involved in resistance to conventional cytotoxic chemotherapies [18], we assessed whether CSC markers were upregulated in our population of 5-FU chemoresistant cells. Using reverse transcription-qPCR, CSC markers such as *CD44*, *CD133*, *ALDH1A1*, *NANOG*, *SOX2*, and *POU5F1* (OCT-4) were evaluated. HCT-116 5FUR cells showed a trend to increase POU5F transcript levels (*p* = 0.08) but had a significant increase in the expression of *ALDH1A1* when compared to the HCT-116 control group (Fig. 1d). Acetaldehyde dehydrogenase 1 (ALDH1) is one of the essential markers in CSCs. The expression level of *ALDH1* in solid tumor tissues, including the colorectal cancer, is higher than in normal tissues, and in recent years, the possibility of *ALDH1* as a potential therapeutic target for CSCs has been discussed [19, 20]. A recent study of Babajnai et al. [21] showed that CRC cells resistant to 5-FU displayed upregulation of CSC genes, EMT-related genes, and downregulation of E-cadherin, compared to parental cells. In addition, increased expression of *ABC* transporter genes was also observed, suggesting their associations with genes related to CSC phenotype, which corroborates with our findings. Other study showed that CSCs enrichment after 5-FU treatment also contribute for apoptosis evasion and cell cycle modulation. Indeed, CSCs are poorly differentiated and with distinct characteristics such as quiescent state, metabolic switch, aberrant activation of different signaling pathways, and resistance to DNA damage, which explain the cell survival and tumor recurrence after cytotoxic treatments [22].

### The acquisition of chemoresistance to 5-FU increased Wnt/β-catenin activation in CRC cells

**Fig. 2:**
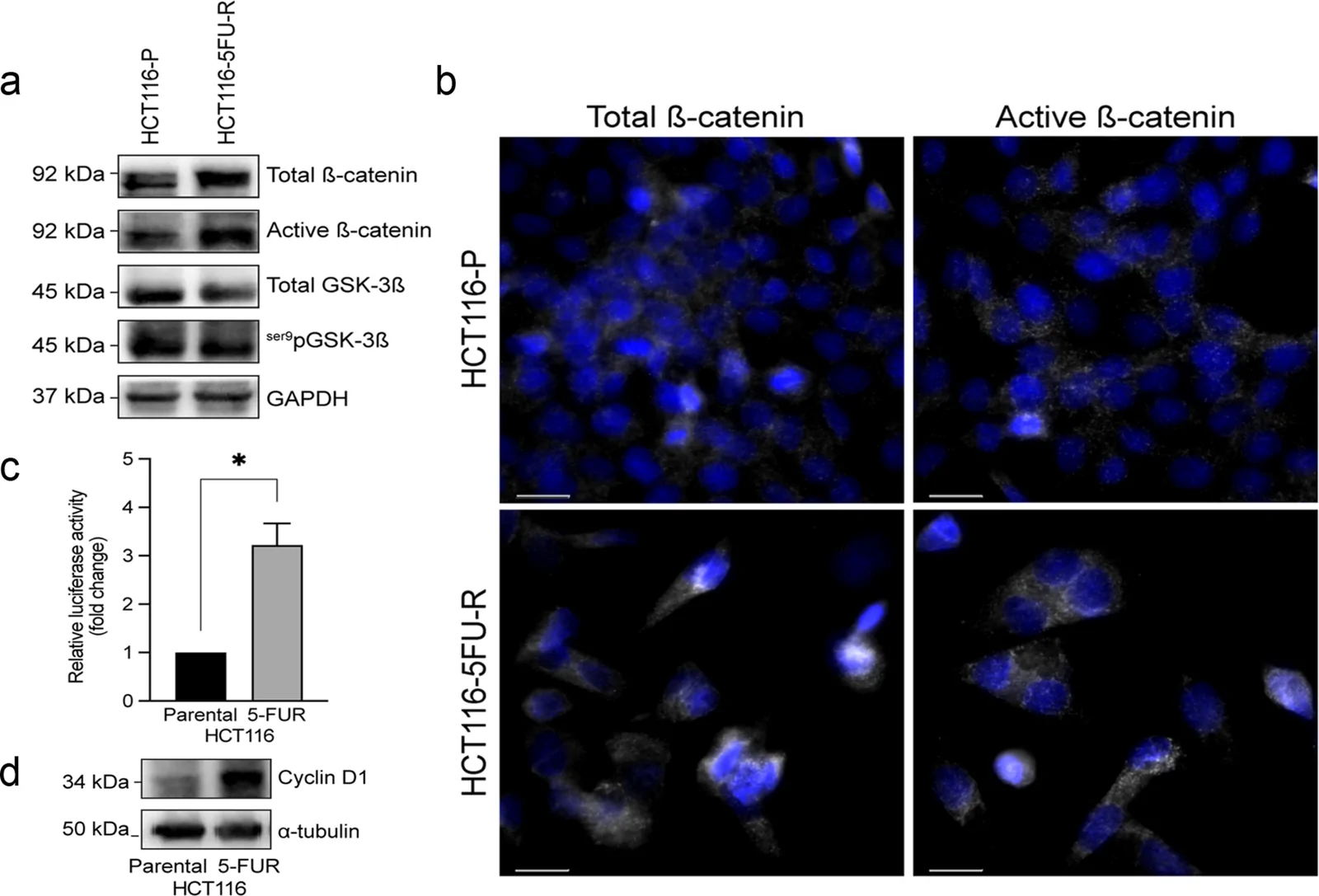
Characterization of long-term 5-FU‐resistant HCT-116 (HCT-116 5FUR) cells in relation to Wnt/β catenin pathway activation (**a**) Western blot of the canonical Wnt/β catenin pathway components. Representative data from three independent experiments for total and active β catenin, and two experiments for total GSK 3β and GSK 3β ser9. (**b**) Evaluation of the subcellular localization of total β catenin and active β catenin (grayscale) using immunofluorescence. Nucleus stained with DAPI (blue). Scale bar: 100 μm. (**c**) Luciferase assay TCF/LEF in HCT 116 control and HCT 116 5FUR cells. Data are representative of the mean ± SEM of three independent experiments. Statistical analysis was performed using Student’s t-test. The difference was considered significant when *P < 0.05. (**d**) Cyclin D1 protein levels evaluated using western blot in HCT 116P and HCT 116 5FUR cells. Representative data from three independent experiments

Next, we assessed the Wnt/β-catenin activation status by analyzing the phosphorylation of downstream components of this pathway. We observed increased protein level of total and active (dephosphorylated) β-catenin in HCT-116 5FUR cells in relation to those in the control group. The dephosphorylated form of β-catenin corresponds to the active form of this protein, which accumulates in the cytoplasm and is subsequently translocated to the nucleus, where together with the TCF/LEF complex, it initiates the transcription of target genes [23]. Additionally, a decrease in the total levels of the enzyme GSK-3β was observed in HCT-116 5FU resistant cells compared to those of the control group, and no differences were observed in the phosphorylated GSK-3β at serine 9 (inactive form) in both the groups (Fig. 2a). These results were corroborated by immunofluorescence imaging where increased total and active β-catenin staining in the nucleus and cytoplasm was observed in HCT-116 5FUR cells, compared to that in the control cells (Fig. 2b). Subsequently, we used the TCF/LEF Luciferase assay to confirm Wnt/β-catenin via activation. We observed increased transcriptional activity of β-catenin in the HCT-116 5FUR cells in relation to that of the control group (Fig. 2c). Moreover, levels of the cyclin D1, a cell cycle marker and well-known downstream target of the Wnt/β-catenin was increased in the HCT-116 5FUR group (Fig. 2). In summary, these data demonstrate that the acquisition of 5-FU chemoresistance in HCT-116 cells can induce increased Wnt/β-catenin activation in CRC cells. Wnt signal activation induced by 5-FU resistance was also observed in TP53 wild-type CRC cells, HCT-8, suggesting a crosstalk between the Wnt pathway and the p53-regulated apoptotic pathway during drug resistance [24]. Furthermore, p53 promoted *WNT3* transcription, leading to WNT/β-catenin pathway activation in ApcMin/+/Lgr5EGFP mice, CRC patient-derived tumor organoids and patient-derived tumor cells. Through this regulation, 5-FU induced activation of this via and CSCs enrichment in the residual tumors, contributing to recurrence [25]. Thus, suggesting that p53 is an important mediator of CSC activation 5-FU-induced through Wnt/β-catenin signaling.

### Inhibition of the Wnt/β-catenin pathway with LF3 sensitizes and potentializes its effect on HCT-116 5FUR cell viability

**Fig. 3:**
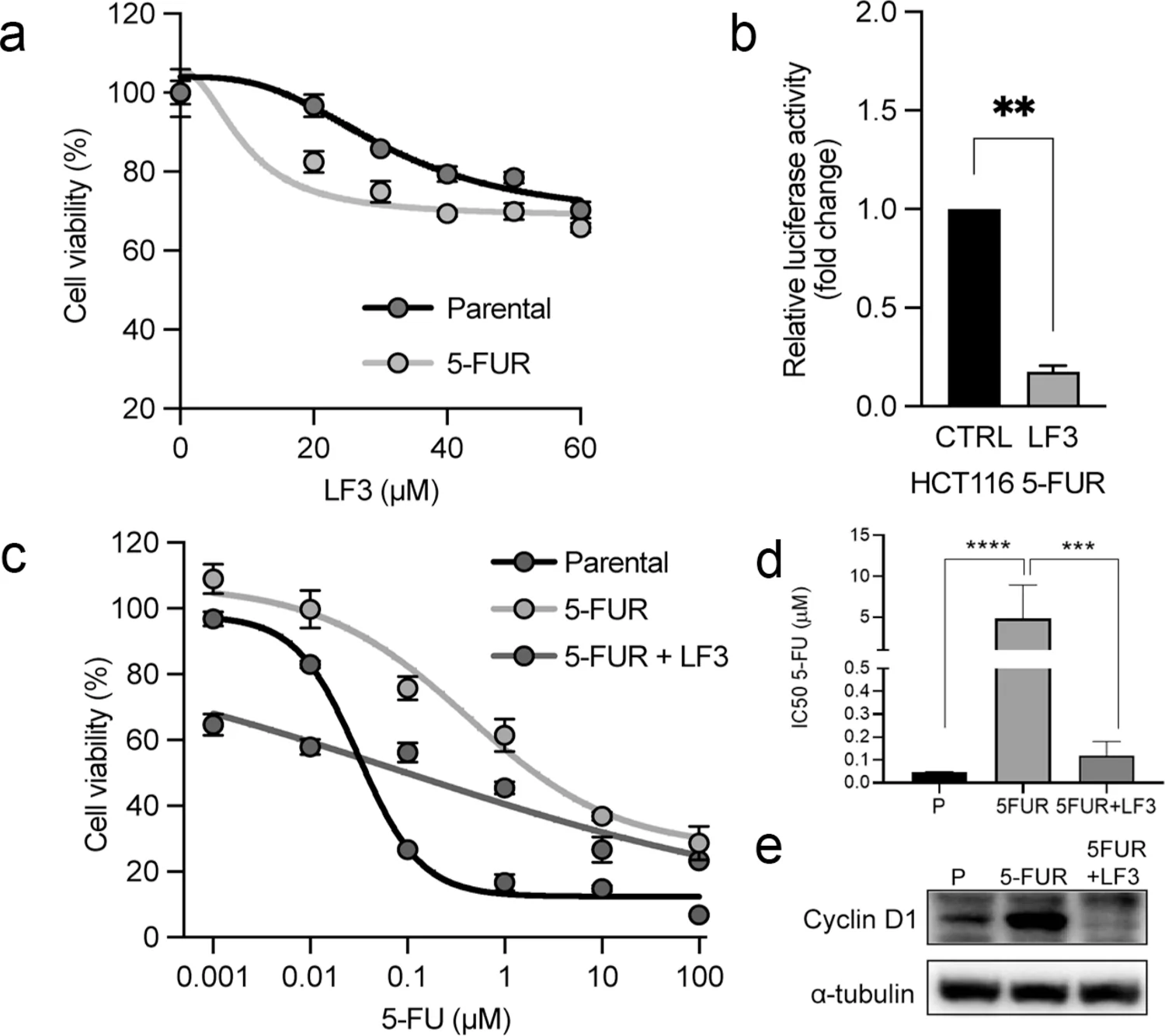
Effect of the LF3 inhibitor on Wnt/β catenin pathway activation (**a**) Determination of cell viability in HCT 116 control and HCT 116 5FUR cells using the MTT assay after treatment for 48 h with 60 µM LF3. (**b**) Transcriptional activity analysis via the TCF/LEF luciferase assay in HCT 116 5FUR cells following treatment with 60 µM LF3. Statistical analysis was performed using Student’s t-test. The difference was considered significant when **P < 0.01. (**c**) Effect of LF3 addition on the cell viability and IC_50_ value for 5-FU (**d**) of 5-FU in HCT-116 5FUR cells after 48 h of treatment. Statistical analysis was performed using one-way analysis of variance followed by Tukey’s post-test. For both tests, the difference was significant when ***P < 0.001, ****P < 0.0001, (**e**) Western blot evaluation of cyclin D1 following treatment with LF3 for 24 h in HCT 116 5FUR cells. Data are representative of three independent experiments

The canonical Wnt/β-catenin pathway activation can be inhibited by targeting its various upstream and downstream components with conventional and repurposed drugs as well as natural compounds selected based on preclinical and clinical studies, as previously proposed for CRC, using the LF3 inhibitor [8]. Thus, to further confirm Wnt/β-catenin pathway activation during resistance acquisition in our previously described 5-FU-resistant model, we pharmacologically inhibited this pathway using LF3. LF3 is a 4-thiourea benzenesulfonamide derivative known as a strong inhibitor of the β-catenin/TCF4 interaction. The sulfonamide group of LF3, being negatively charged binds to Lys435 on β-catenin, competitively inhibiting its binding to Asp16 in TCF4, thus blocking the Wnt signaling pathway [26]. LF3 inhibits Wnt/β-catenin in cells with exogenous reporters and in colon cancer cells with endogenously high Wnt activity. LF3 also suppresses high cell motility, cell-cycle progression, and Wnt target genes overexpression, which are related to Wnt signaling [27]. This pharmacological approach was chosen to avoid compensatory upstream activation of the pathway since frequent mutations in *APC*, *RNF43*, and *AXIN2*, which have been reported in CRC, would result in truncated protein formation and constitutive activation of the Wnt/β-catenin pathway [28]. First, to validate the use of LF3 as a pathway inhibitor, we analyzed the effect of LF3 treatment on cell viability using the MTT assay. After the 48-h treatment, we observed approximately a decrease of 30% of the cell viability in both groups at the maximum LF3 concentration tested (60 µM) (Fig. 3a). Furthermore, IC_50_ values of 103.5 µM and 135.2 µM were obtained for HCT-116 control and HCT-116 5FUR cells, respectively. Thus, 60 µM LF3 did not exert extensive cytotoxic effects on CRC cells. Next, the TCF/LEF luciferase assay was used to confirm the inhibitory effect of LF3 on the Wnt/β-catenin signaling pathway. HCT116-5FU- resistant cells were treated with 60 µM LF3 for 24 h. Approximately six-fold decrease in the relative luciferase activity was observed in the LF3-treated HCT-116-5FUR group compared to that in untreated HCT-116-5FUR cells (Fig. 3b). Therefore, LF3 effectively inhibited the Wnt/β-catenin pathway at the evaluated time. Subsequently, we analyzed the effect of the combined treatment of 5-FU and LF3 on HCT1165FU-resistant cells. The cell viability curve obtained for the MTT assay showed that HCT-116 5FUR cells exhibited higher cell viability and IC_50_ (4.8 µM) values than those of the control group (0.035 µM). However, adding the LF3 inhibitor to HCT-116 5FUR cells further decreased cell viability and IC_50_ for 5-FU (0.138 µM) relative to that on treatment with 5-FU alone (Figs. 3c, d). Finally, the LF3 effect on the levels of cyclin D1 was also analyzed. Treatment with 60 µM LF3 for 24 h reduced cyclin D1 protein levels (Fig. 3e). Collectively, our results indicate that inhibition of the Wnt/β-catenin pathway with LF3 can sensitize our model of HCT-116 5FU resistant cells to 5-FU treatment and potentiate the effect decreasing the viability of chemoresistant CRC cells. Studies have highlighted the significance of combinatorial treatment of Wnt signaling inhibitors and 5FU as a better treatment strategy to overcome 5FU resistance. An early study showed that LF3, an antagonist of the β-Catenin/TCF4 interaction, blocked the self-renewal of CSCs and suppresses tumorigenesis in a CRC model, suggesting LF3 as a specific inhibitor of the canonical Wnt signaling with anticancer activity [25]. More recently, was investigated the synergistic cytotoxic activity of 5FU with menadione, a small molecule that downregulate Wnt signaling in CRC cells. Results showed that the simultaneous binding of 5FU and menadione to β-catenin can block Wnt signaling by disrupting β-catenin-TCF interaction, inhibiting the proliferation of CRC cells [29].

## Conclusions

Our results show that CRC cells resistant to 5-FU (HCT-116 5FUR) exhibited significant increase in the expression of *ALDH1A1*, a classical CSC marker, and high Wnt/β-catenin pathway activity, decreasing the efficacy of 5-FU treatment. Importantly, the pharmacological inhibition of the Wnt/β-catenin pathway with LF3 potentiated the cytotoxic effect of 5-FU, impairing the chemoresistant phenotype of CRC cells. Since our previous results using this same cellular model showed that the 5-FU resistant cells developed EMT features [14], future studies are warranted to identify if these potential chemoresistance mechanisms such as EMT and stemness are interconnected or associated with the Wnt/β-catenin pathway. It is known that complete loss of epithelial features with a total mesenchymal phenotype gain during EMT is rare in human carcinomas. What really happens is that cells in EMT display intermediate phenotypic states, which seem be responsible for the development of a tumor stem phenotype, inducing mechanisms of therapeutic resistance. In this context, recently we hypothesize that cells in partial EMT could also originate stemness characteristics through activation the canonical Wnt/β-catenin pathway. Thus, considering that the activation of this pathway can be inhibited by targeting different upstream and downstream components its inhibition could overcome therapeutic resistance and exert anticancer activity by suppressing stemness in therapy-induced EMT cells [30]. Therefore, druggable experimental cellular models to induce 5-FU long-term resistance, as our model, can allow the monitoring and characterizing these two cellular phenotypes in future preclinical studies to refine therapeutic strategies for improving therapeutic effects and cost-effectiveness in CRC treatment.

## Data Availability

Enquiries about data availability should be directed to the authors. The data that support the findings of this study are available from the corresponding author upon reasonable request.
